# Cryo‐FIB preparation of whole cells and tissue for cryo‐TEM: use of high‐pressure frozen specimens in tubes and planchets

**DOI:** 10.1111/jmi.12943

**Published:** 2020-07-28

**Authors:** D.A.M. DE WINTER, C. HSIEH, M. MARKO, M. F. HAYLES

**Affiliations:** ^1^ Environmental Hydrogeology, Department of Earth Sciences Utrecht University Princetonlaan 8a Utrecht the Netherlands; ^2^ New York State Department of Health Wadsworth Center, Empire State Plaza Albany New York U.S.A.; ^3^ College of Nanoscale Science and Engineering SUNY Polytechnic Institute Albany New York U.S.A.; ^4^ Cryo‐FIB‐SEM Technologist Eindhoven the Netherlands

**Keywords:** Cryo, focused ion beam‐scanning electron microscope, high‐pressure freezing, lamellae, life sciences, transmission electron microscope

## Abstract

The desire to study macromolecular complexes within their cellular context requires the ability to produce thin samples suitable for cryo‐TEM (cryo‐transmission electron microscope) investigations. In this paper, we discuss two similar approaches, which were developed independently in Utrecht (the Netherlands) and Albany (USA). The methods are particularly suitable for both tissue samples and cell suspensions prepared by a high‐pressure freezer (HPF). The workflows are explained with particular attention to potential pitfalls, while underlying principles are highlighted (‘why to do so’). Although both workflows function with a high success rate, full execution requires considerable experience and remains demanding. In addition, throughput is low. We hope to encourage other research groups worldwide to take on the challenge of improving the HPF– cryo‐FIB‐SEM – cryo‐TEM workflow. We discuss a number of suggestions to this end.

**Lay Description:**

Life is ultimately dictated by the interaction of molecules in our bodies. Highly complex equipment is being used and further developed to study these interactions. The present paper describes methods to prepare small, very thin lamellae (area of 5×5 µm^2^, thickness 50–300 nm) of a cell to be studied in a cryo‐transmission electron microscope (cryo‐TEM). Special care must be taken to preserve the natural state of molecules in their natural environment. In the case of cryo‐TEM, the samples must be frozen and kept frozen to be compatible with the vacuum conditions in the microscope. The frozen condition imposes technical challenges which are addressed. Two approaches to obtain the thin lamellae are described. Both make use of a focused ion beam (FIB) microscope. The FIB allows removal of material with nanometre precision by focusing a beam of ionised atoms (gallium ions) onto the sample. Careful control of the FIB allows cutting out of the required thin lamellae. In both strategies, the thin lamellae remain attached to the original sample, and the ensemble of sample with section and sample holder is transported from the FIB microscope to the TEM while being kept frozen.

## Introduction

The study of molecules *in situ* –within the cellular context– and at the macromolecular level has long been the ambition of electron microscopy applied in biology. While electron optics and advanced image‐processing techniques have progressed into the subnanometre spatial‐resolution range (Asano *et al*., 2016), major challenges remain in the field of sample preparation. Making specimens that are compatible with the TEM (transmission electron microscope) vacuum affects the biological ultrastructure to a certain extent, depending on the preparation method (Kellenberger *et al*., 1992; Zechmann *et al*., 2006; Hunziker *et al*., 2014). Freezing is considered to be the best choice to maintain the native state of the specimen (Studer *et al*., 2008; Mielanczyk *et al*., 2014). Vitreous freezing, without dehydration or staining, allows the highest possible resolution of macromolecules (Doerr, 2015).

A variety of techniques can be employed to freeze a specimen in vitreous (noncrystalline) ice. Vitreous freezing is necessary because ice‐crystal formation generates mechanical forces, osmotic pressure differences and phase segregation, all of which interfere with cellular ultrastructure. For a single layer of cells, rapid freezing in liquid propane or ethane is successfully employed. Thicker specimens can be placed inside a metal tube or planchet and frozen by a high‐pressure freezer (HPF). Small volumes of hydrated cellular material (up to 1 mm^3^) can be vitreously frozen at high pressure, thus preventing ice‐crystal formation (Studer *et al*., 1995). After freezing, the temperature must be maintained below –140°C to prevent recrystallisation of the vitreous ice, which imposes constraints on sample handling.

High‐resolution cryo‐TEM imaging of macromolecules *in situ* requires samples not exceeding a thickness of about one inelastic mean‐free path for electrons (∼200–400 nm, depending on accelerating voltage). Engineering challenges to prepare such samples have been met in recent years, which will be discussed, but much work is still needed. A breakthrough was made in 2006, when it was shown that a focused ion beam (FIB) – integrated into a cryo‐scanning electron microscope (cryo‐SEM) – can thin ice samples without affecting the vitreous state of the specimen (Marko *et al*., 2006, 2007). Since 2006, three approaches have emerged. (1) Cells or bacteria frozen on a TEM grid – the cryo‐FIB thins areas of interest for cryo‐TEM (Marko *et al*., 2006; Marko *et al*., 2007; Rigort *et al*., 2010; Rigort *et al*., 2012; Strunk *et al*., 2012; Zhang *et al*., 2016; Schaffer *et al*., 2017); (2) the ‘lift‐out’ technique, known from the semiconductor industry and material sciences (Mayer *et al*., 2007), but carried out under cryo‐conditions (Rubino *et al*., 2012; Mahamid *et al*., 2015; Parmenter *et al*., 2016; Schaffer *et al*., 2019; Kuba *et al*., 2020; Parmenter & Nizamudeen, 2020) and (3) freezing the specimen in a tube or planchet (also known as ‘freezing hat’ or ‘platelet’) – the cryo‐FIB thins a region of the specimen using the ‘H‐bar’ technique (Edwards *et al*., 2009; Hayles *et al*., 2010; Hsieh *et al*., 2014). The advantage of using planchets or tubes is the potential to investigate cell–cell contacts or specific organelles within cells in the context of surrounding cells, and in particular within tissue. A crucial step is using a correlative light microscopy approach in order to locate a specific feature of interest (Rigort *et al*., 2012). Methods (1) and (2) are discussed elsewhere in this special issue. The present paper will discuss the practicalities, advantages, disadvantages and near‐future challenges of method (3). Very similar procedures have been developed in Utrecht (the Netherlands) (Hayles *et al*., 2010) and Albany (NY, US) (Hsieh *et al*., 2014); details from both will be discussed in the following sections.

### Main challenges

The H‐bar approach avoids direct handling of the TEM lamellae, while enabling the use of HPF‐prepared specimens. Therefore, the specimens require a carrier that can be easily handled without touching the specimen directly, and that holds the specimen throughout the entire process. General challenges are: (1) milling TEM‐thin lamellae in such a way that they can be imaged in a tomographic tilt series and (2) avoiding frosting or devitrification, especially post‐FIB‐milling. Here, we describe methods to address these challenges.

### Procedure in brief

**Fig. 1:**
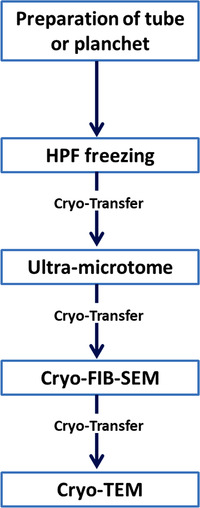
Flow chart of the entire process of cryo‐FIB prepartion. All the cryo‐transfer steps are critical in terms of warming, frost contamination, and physical damage. Therefore, reducing direct sample handling improves the success rate.

The general workflow is shown in Figure 1. Once frozen, the specimens are either kept in liquid nitrogen, in dry nitrogen gas for a very short moment in time, or are actively cooled within a vacuum. These measures are required to prevent devitrification and the condensation of moisture from air onto the specimen. Moisture condensation is further reduced by keeping all the ports used for the cryo‐transfer procedure under vacuum. When using the ports (see ‘Processing high‐pressure‐frozen samples’ section), dry nitrogen gas is used for venting.

**Fig. 2:**
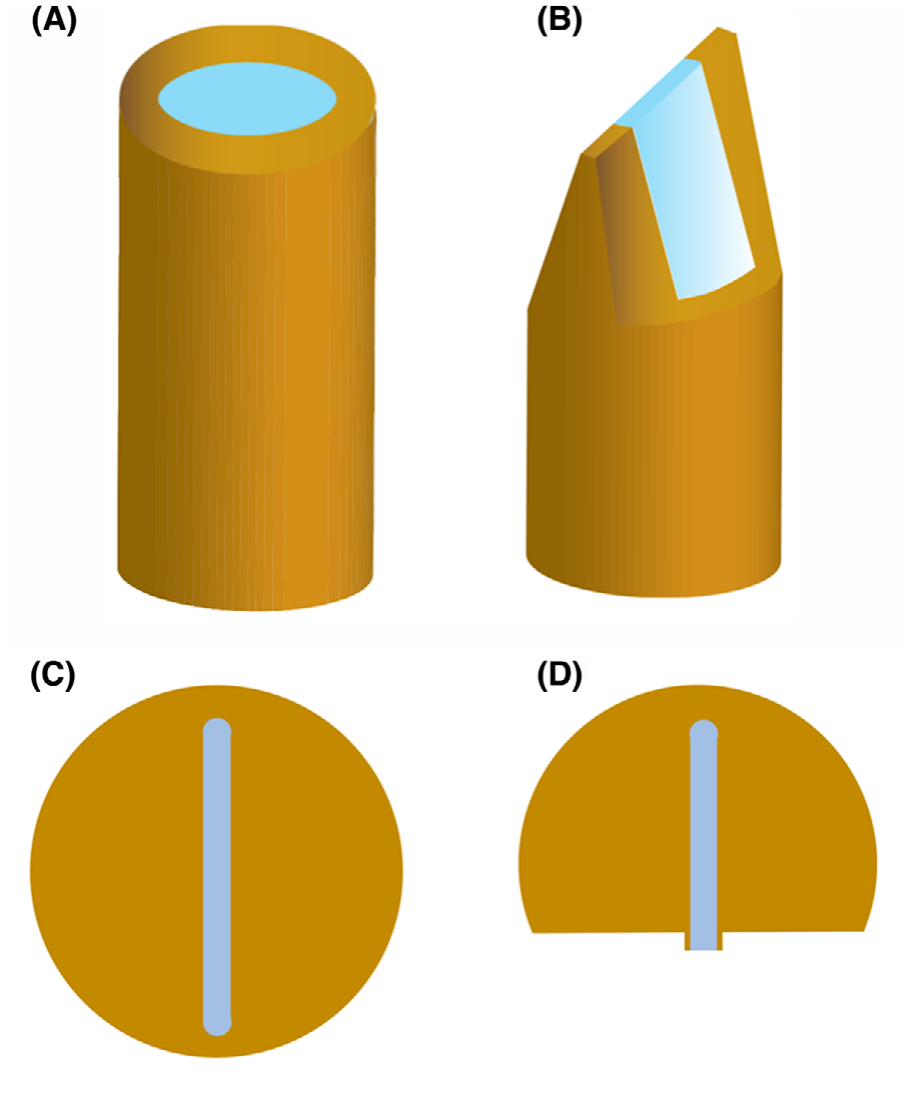
Schematic of trimming high‐pressure‐frozen samples in the cryo‐ultramicrotome. (A), (B) Specimens frozen in copper tubes (inner diameter 0.35 mm); excess metal is removed in a cryomicrotome leaving a wedge‐shaped (‘rooftop’) geometry that facilitates fast and efficient FIB‐milling. (C), (D) Specimens frozen in standard aluminium planchets (‘hats’); metal is similarly removed, exposing the specimen supported by residual aluminium, and then the specimen thickness at the edge is reduced to 20 µm.

The metal casing of the tube/planchet is cut away (e.g. by cryo‐ultramicrotomy) in order to expose the specimen within, as shown in Figure 2. As a result of this process, only a limited amount of thinning by FIB milling is required, greatly reducing the processing time. In the case of the tube, the wedge‐shaped geometry also helps to enable imaging at high tilt in the TEM. After trimming with a cryo‐ultramicrotome, the tube/planchet is transferred into the FIB‐SEM. Finally, transfer is made from the cryo‐FIB‐SEM into the cryo‐TEM.

The entire procedure from cryo‐ultramicrotome trimming to cryo‐TEM imaging can be performed within one day, which can limit the chance of contamination on the specimen from overnight storage and related additional transfers. Sometimes the specimen is stored under liquid nitrogen between steps in the process, although ice particles in the liquid nitrogen may deposit on the specimen over time, which is bothersome if the specimen has already been thinned for cryo‐TEM. An alternative might be to keep the specimens in the cryo‐FIB‐SEM chamber overnight at a prefinal stage, provided that the cryo‐system can maintain its temperatures without supervision. Still, a thin layer of ice may condensate on the surface from residual water vapour in the vacuum chamber (Weikusat *et al*., 2011; Schaffer *et al*., 2017).Therefore, it is recommended to perform the final thinning steps shortly before making the transfer to the cryo‐TEM. A general measure to reduce the water vapour deposition in the cryo‐FIB‐SEM chamber is installing the cryo‐stage the day before the experiment. Overnight pumping will remove most of the water vapour, but some ice contamination (condensation) is inevitable, even with an anticontamination surface in the FIB‐SEM vacuum chamber.

## Materials and methods

### Specimen

#### High‐pressure freezing

Freezing cells and tissues is a particularly delicate exercise. The poor thermal conductivity of ice limits the freezing rate in bulk samples, so high freezing rates are required to prevent ice‐crystal formations. Therefore, cells and tissues are not always high‐pressure frozen ‘as is’. Some substance is usually added, which functions as a ‘filler’ or ‘cryo‐protectant’, and ideally both (McDonald, 2007; Möbius *et al*., 2010; Mielanczyk *et al*., 2014). A filler is used to avoid imperfect and inhomogeneous freezing from voids of air, while a cryo‐protectant binds to water molecules, preventing ice crystallisation (Hayles & De Winter, 2020).

#### High‐pressure freezing cells (Utrecht)

Fresh baker's yeast cells (*Saccharomyces cerevisiae*) are centrifuged into a soft pellet after being cultured in a standard culture medium and subsequently mixed with 20% Dextran (40 kDa; Sigma # 31389) as filler. The mixture of yeast cells and Dextran is pushed and pulled through Leica EM PACT copper tubes several times before freezing in the EM PACT (Leica Microsystems, Vienna, Austria), to ensure complete filling without air spaces. Preparation of yeast cells in planchets follows the method described in detail in De Winter *et al*. (2013). As planchets, 100‐µm‐deep Leica membrane carriers are used. High‐pressure freezing is also carried out in an EM PACT unit.

#### High‐pressure freezing tissue (Albany)

Zebrafish muscle tissue is lightly fixed in glutaraldehyde to improve the handling characteristics and is cut into small, thin pieces to fit standard 3 mm Leica slot‐type planchets. Hexadecane is used to fill any air gaps in the slot. Specimens are frozen in standard, covered, slot‐type planchets (‘hats’) provided for the HPM‐010 high‐pressure freezer (now produced by ABRA Fluid AG, Widnau, Switzerland). The assembled hats are stored under liquid nitrogen for later use.

### Processing high‐pressure‐frozen samples

#### Planchets and tubes (Utrecht)

**Fig. 3:**
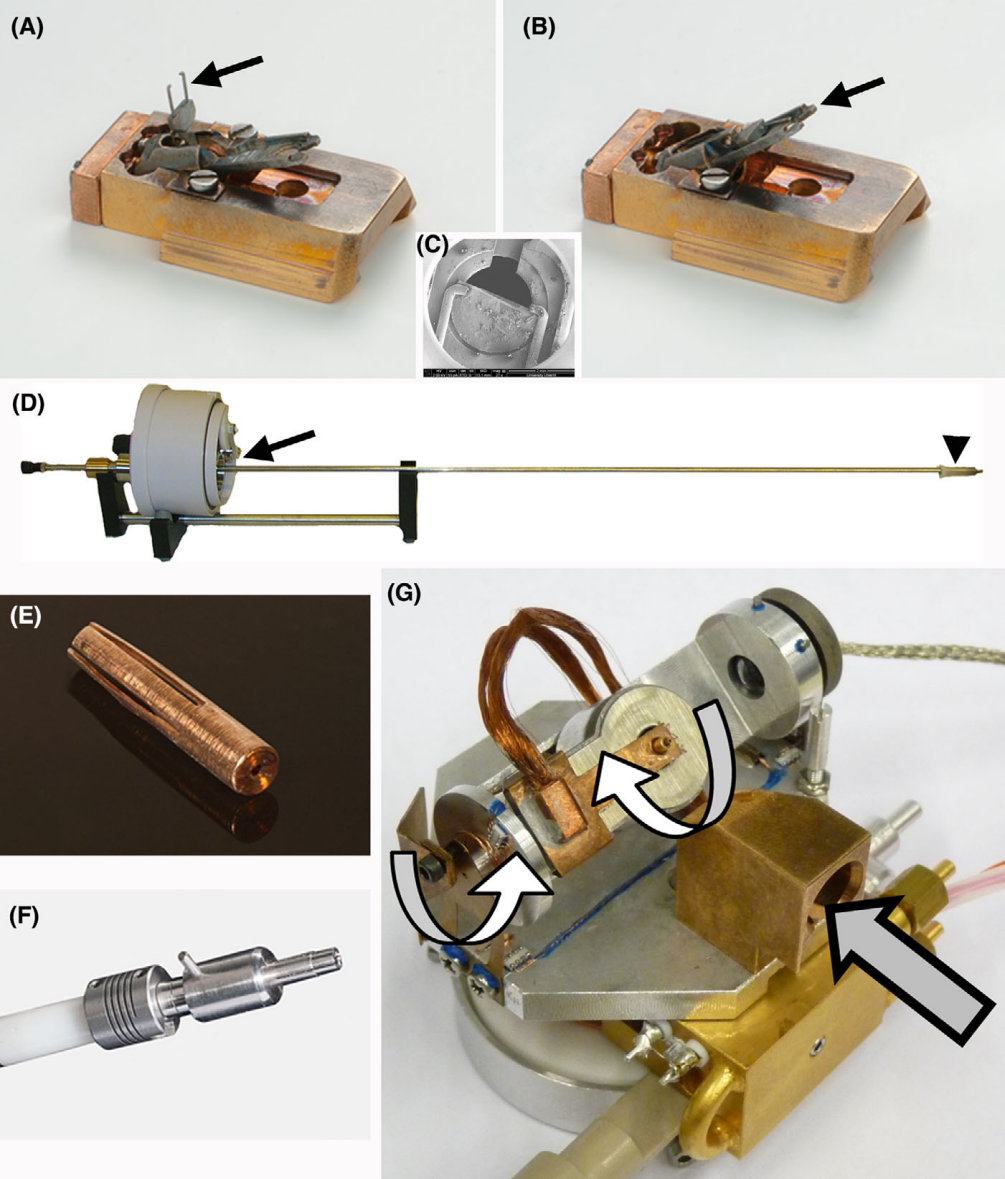
Utrecht methods for handling samples in the cryo‐FIB (after FIB‐milling, samples are transferred to a modified TEM cryo‐transfer holder, not shown here). (A)–(D) Handling planchettes or TEM grids. (A) The cryo‐holder (slegde) is modified to hold a modified TEM specimen holder, with the clamp indicated by an arrow in (A); the holder can be flipped up to 38°, as shown by the arrow in (B), for access to the FIB beam via a slot made at the end of the a TEM specimen‐stage sample ‘clip’, as shown in (C), the sample having already been trimmed in the cryo‐ultramicrotome. Cryo‐transfer of the sledge (or the ferrule transfer device) is done with the Quorum transfer module (D), which has a sealed chamber (at the arrowhead) which protects the sample under vacuum during transfer. (E)–(G) Handling specimens frozen in tubes. The tube with the specimen is loaded into the ferrule (E) after high‐pressure freezing to circumvent manual handling of the tube. The splined end of the ferrule is spring‐loaded to hold the ferrule in place inside the accepting hole. The transfer chuck (F) is screwed to the end of the transfer rod and can pick up and release the ferrule from the transfer module and the cryo‐nano bench (CNB) (G). The CNB itself has two rotation axes (white arrows), which compliment the standard FIB‐SEM stage movements. The splined end of the ferrule is guided through a block in the CNB (grey arrow) into the accepting hole, and the chuck is then removed. After FIB‐milling, the chuck is used to transfer the ferrule (with its copper tube), by means of the Quorum transfer modue, to a modfied TEM cryo‐transfer workstation not shown), inside of which the copper tube is inserted into the TEM holder.

After freezing, trimming is done in a UCT/FC cryo‐ultramicrotome (Leica Microsystems, Vienna, Austria), to uncover the frozen specimen. During trimming, the planchets are held in a custom fixture (De Winter *et al*., 2013), while copper tubes are held in a custom ferrule (Hayles *et al*., 2010). After trimming in the cryo‐ultramicrotome, the planchet is loaded into a modified side‐entry TEM specimen holder, which is mounted onto a modified cryo‐transfer sledge (Figs. 3A and B). The planchet is held in place by a clip in a similar fashion as regular TEM grids, as shown in Figure 3(C).

Cryo‐FIB‐SEM milling is done in a Nova Nanolab 600 (Thermo‐Fisher Scientific, Eindhoven, NL, USA) equipped with a PP2000 cryo‐system (Quorum Technologies, Laughton, UK) mounted to the FIB‐SEM specimen chamber. The PP2000 allows transfer of a prefrozen specimen into the microscope via its ‘Advanced Transfer Unit’ (ATU), which is filled with liquid nitrogen and has a small stage that accommodates the sledge. The sledge is picked up by the rod of the transfer device (Fig. 3D). Before retracting the rod, the ATU is pumped down to establish a vacuum. The sledge is lifted above the LN2 (liquid nitrogen) level just before the LN2 solidifies. The transfer device is released from the ATU and then attached to the PP2000 preparation chamber. The sledge can be placed in the preparation chamber once the space in between the transfer device and the preparation chamber is pumped down. Inside the preparation chamber, the sample is actively cooled, and a metal film is applied by sputter coating. Before subsequent transfer onto the FIB‐SEM cryo‐stage, the planchet holder can be tilted to a 38° position (Fig. 3B) using the manual handling tool in the preparation chamber. Once the temperature between the cryo‐prep chamber and the FIB‐SEM cryo‐stage is balanced by raising the temperature of the cryo‐prep chamber, the transfer rod is used to slide the sledge onto the cryo‐stage. Balancing the temperatures ensures that when the sledge enters the FIB‐SEM chamber, the sledge is not the coldest surface in the FIB‐SEM chamber. The sledge would become the preferred surface for condensation of water vapour left in the chamber when it is the coldest object in the chamber, thereby potentially contaminating the sample. A temperature of –150°C for the sample is considered optimal. It is safely below the devitrification temperature of –138°C, while the anticontaminator can be run 20°C colder to reduce the partial water vapour pressure below the condensation conditions for the sample itself (Weikusat *et al*., 2011).

In order to accommodate the copper tubes, a special ferrule (Fig. 3E) was developed to hold the tube during the whole process, which starts with trimming in the cryo‐ultramicrotome. The ferrule, in turn, fits into a transfer chuck operated by a screw mechanism (Fig. 3F), which fits into the end of the rod of the transfer device, and which is used along with a special cryo‐stage called the ‘Cryo‐Nano Bench’ (CNB) (Fig. 3G) (Hayles *et al*., 2010). The CNB is mounted on top of the standard FIB‐SEM stage. A guiding block on the CNB leads the ferrule into the accepting hole of the CNB, after which the ferrule is released from the transfer chuck. The CNB is equipped with two piezo‐driven motors that allow continuous rotation around the ferrule's longitudinal axis, and tilting from the horizontal position to the required 38° for FIB milling. The standard FIB‐SEM stage provides lateral movement and allows for height changes. The additional axes provided by the CNB greatly improve versatility and also facilitates improved SEM inspection of the milling results.

After FIB‐milling (see ‘Cryo‐FIB milling procedures’ section), the transfer chuck, holding the ferrule, is cryo‐transferred out of the FIB‐SEM using the ATU. Then the chuck is directly inserted into a Gatan Model 626 cryo‐TEM workstation (Gatan, Warrington, PA, USA), which was specially modified (Hayles *et al*., 2010). Inside the workstation, a special mechanism releases the ferrule and inserts it into the modified TEM cryo‐transfer holder. In this way, the user never needs to handle the ferrule directly, nor even the copper tube. In the case of planchets, manual handling with tweezers is required. The sledge is released in LN2 in the ATU and transferred in a small LN2‐containing cup to the TEM cryotransfer workstation. The working station is filled with LN2 as well, as the planchet is released from the sledge and placed in the cryo‐TEM specimen holder. Cryo‐TEM images are recorded in a Tecnai‐12 (Thermo‐Fisher Scientific, Eindhoven, the Netherlands).

#### Planchets (Albany)

As at Utrecht, the goal at Albany is to avoid direct handling of the specimen carrier used in the high‐pressure freezer, thus protecting the delicate sample at all times, and especially after FIB‐milling.

**Fig. 4:**
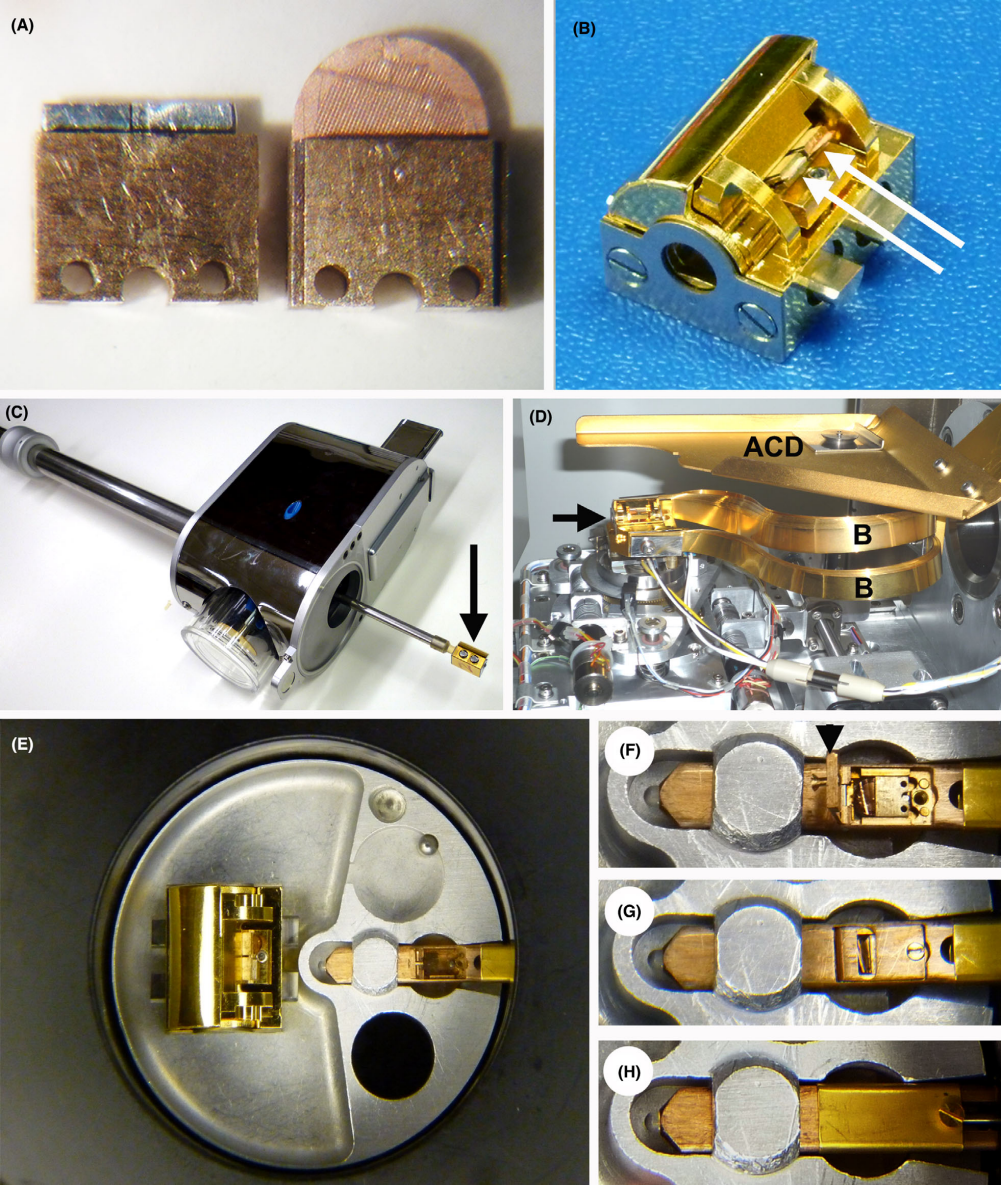
Albany methods for handling specimens in the cryo‐FIB. Planchets or intact TEM grids are held in a clamp (A); the clamp also fits the cryo‐ultramicrotome for pretrimming the planchettes. The clamps (arrows) are placed in a custom Leica cryotransfer block (B) having a half‐cylinder shutter that is closed when the block (arrow) is placed in the Leica shuttle (C) during cryo‐transfer, where it is actively cooled and under vacuum. The cryo‐FIB stage (D) accepts the cryo‐transfer block (arrow). The stage is cooled by conduction bands (*B*) and has an anticontaminator (*ACD*). After FIB‐milling, the shuttle transfers the block to liquid nitrogen storage. For TEM, the block is opened in the TEM cryo‐transfer workstation (E), and the clamp is placed in a modified TEM cryo‐transfer holder (F), and secured by closing the cover (G). Finally the shutter is closed (H) for TEM cryo‐transfer. Portions of this figure (A, B, E, F, G, H) were previously published in Hsieh *et al*. (2014).

The hats are opened in the chamber of a cryo‐ultramicrotome (Leica UCT/EM‐FCS). The planchet is then inserted into a clamp (Fig. 4A), in which it will remain during trimming and for the rest of the process, including cryo‐TEM imaging (Hsieh *et al*., 2014).

**Fig. 5:**
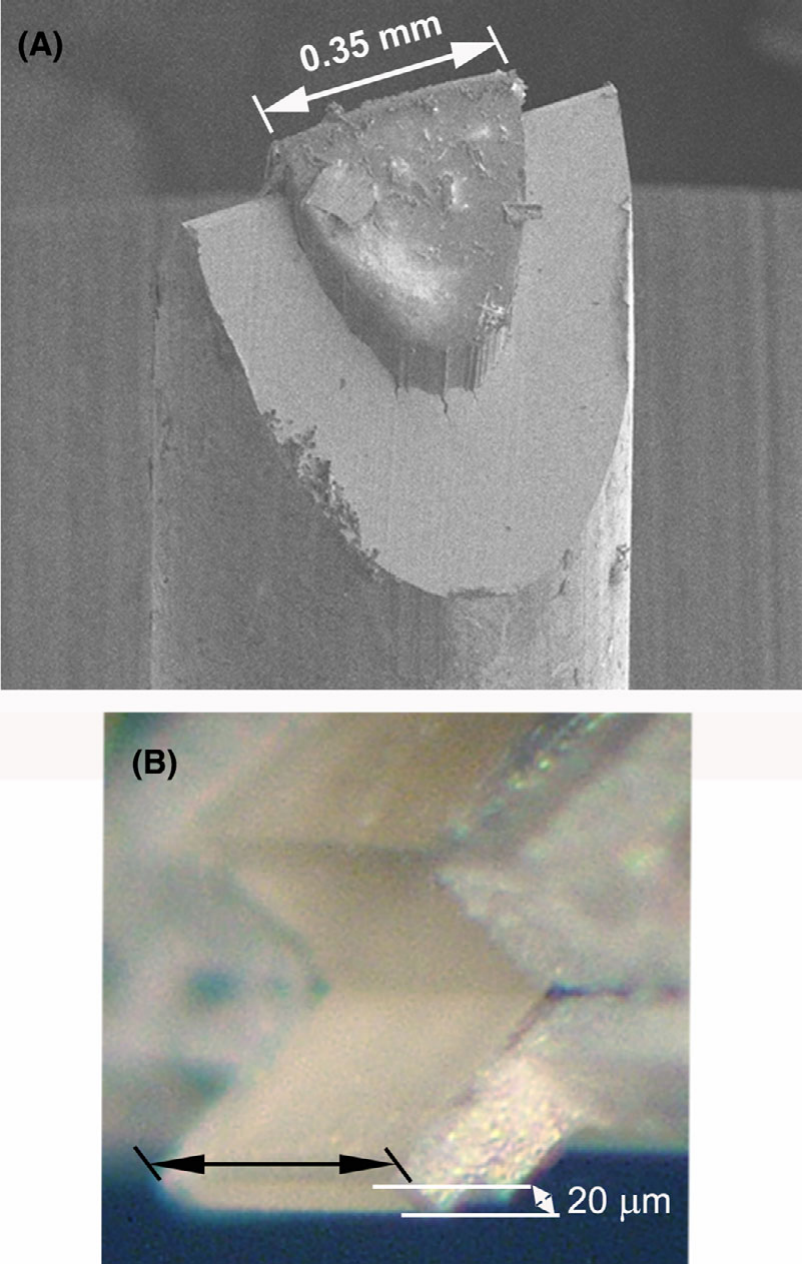
Cryo‐ultramicrotome trimming of high‐pressure‐frozen samples. (A, Utrecht) The wedge‐shaped (‘rooftop’) end of the copper tube after trimming. The sample is forced out of the tube as a result of the temperature difference between the copper tube, still at liquid‐nitrogen temperature after storage, and –160°C inside the microtome chamber. (B, Albany) Planchets are trimmed to expose the edge of the tissue, which is supported at the sides and (slightly away from the edge) by conductive aluminium, which amelioriates charging during cryo‐FIB and cryo‐SEM.

After trimming to expose a thin ridge of tissue (as described later in Fig. 5B), the clamp is mounted in a special pretilted cryo‐transfer block (Fig. 4B) (Leica part number 16770266) that fits in a Leica VCT‐100 cryo‐transfer shuttle (Fig. 4C), and also in the Leica cryo‐stage in the FIB‐SEM (Fig. 4D). The FIB‐SEM is a Neon‐40 EsB (Carl Zeiss, Oberkochen, Germany). The VCT‐100 shuttle facilitates cryo‐transfer under vacuum and with active cooling. The VCT‐100 shuttle also interfaces with a MED‐020 electron‐beam cryo‐coater (Leica Microsystems, Vienna, Austria) so that a metal coating can be applied before or after FIB‐milling (although so far it has not been found to be necessary).

After FIB‐milling, the cryo‐transfer block is transferred by the shuttle to the VCT‐100 loading box, where it is detached and submerged under liquid nitrogen for subsequent storage until wanted for TEM.

The Leica cryo‐transfer block is transferred from the VCT‐100 loading box into a standard TEM cryo‐transfer workstation (Gatan Model 626), in which the Leica cryo‐transfer block is opened. The clamp is then transferred to the tip of a modified Gatan 626 cryo‐transfer holder (Hsieh *et al*., 2014), as shown in Figures 4(E)–(H).

#### Geometry

In many cases, the cryo‐FIB samples will be destined for cryo‐TEM tomographic tilt‐series collection. This requires that a useable area of the lamella can be imaged through a tilt range of at least 120º. The bulk of the sample carrier and the geometry of the thinned sample itself may restrict the area of sample visible at high tilt. In Utrecht, the wedge‐shape end to rods and planchettes, created by microtoming, subsequently produces lamellae in the plane of the wedge, which affords the possibility to align the planchets in the TEM. Ideally, the tilt axis is perpendicular to the edge of the wedge, in the plane of the lamella. In case of a double tilt holder, the second axis should be parallel to the edge of the wedge. The rods are automatically in the correct orientation, while the planchets should be manually placed in the correct orientation. These considerations also govern the design of modified TEM cryo‐transfer holders or cartridges. For both copper tubes and planchets, not only must the sample carrier be cut away, but bevels also need to be milled along the lamellae so that corners of the bulk sample next to the thinned region will not limit the available imaging area when tilted.

### Cryo‐FIB milling procedures

Milling procedures are very similar at Albany and Utrecht. In Utrecht, milling is preceded by coating with a protective layer of platinum using the gas‐injection system inside the FIB‐SEM (Hayles *et al*., 2007). The CNB proves advantageous for this, as the ‘rooftop’ end can be positioned vertically so that both sides of the wedge can be covered. In some cases, a thin platinum layer is first applied by sputter‐coating in the Quorum preparation chamber, before the sample enters the SEM chamber.

**Fig. 6:**
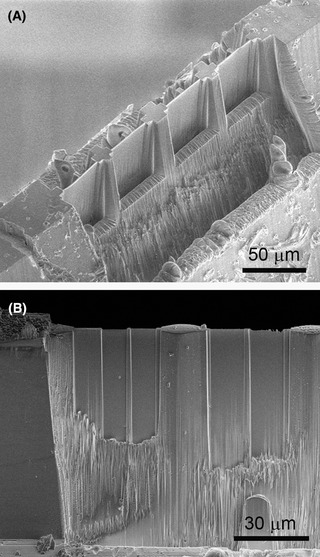
Electron‐transparent windows (‘lamellae’) made by FIB milling [(A) at Utrecht and (B) at Albany]. The width of a lamella is restricted to 10–20 µm by potential flexibility of the thin lamellae,therefore, columns at regular distances are retained for support.

The ‘rooftop’ is forced out of the tube by several tens of micrometres (Fig. 5A) due to a temperature difference between the cryo‐ultramicrotome and liquid nitrogen. This is advantageous, because it reduces the metal of the tube being in the line‐of‐sight of the cryo‐TEM. Moreover, ice is FIB‐milled much more rapidly than silicon or metals (Marko *et al*., 2006; Fu *et al*., 2008), so milling large volumes of ice is not excessively time‐consuming. Using high current (e.g. 20 nA), the full width of the ice sample (0.3–0.35 mm) area can be thinned to 20–30 µm in reasonable time. A faster alternative is using the cryo‐ultramicrotome for thinning the full width (0.3–0.35 mm) of the sample to about 20 µm in thickness (Fig. 5B) (Hsieh *et al*., 2014). Depending on the nature of the specimen and use of cryo‐protectants and fillers, brittleness of the ice may restrict the precision and final thickness of the wedge. After initial FIB‐milling, several lamellae are created, with currents reduced stepwise down to 100 pA for in the final step (Fig. 6). A set of lamellae can be created within a few hours.

First, a ‘FIB‐view’ image is recorded using secondary electrons emitted by interaction of the ion beam with the specimen. Then, a rectangular pattern defining the area to be milled is drawn on the image. Current and milling‐time selection determines the rate and depth of material removal. Successive rectangular patterns are defined, with the current setting decreasing as the sample thickness decreases. The raster characteristics and dwell time are selected after experience with a particular specimen type. On the FEI instrument at Utrecht, the ‘cleaning cross‐section’ mode has proven to be optimal for most work. On the Zeiss instrument at Albany, milling is done by rapid rastering within the rectangular pattern. In both cases, progress is monitored by occasional SEM observation, which conveniently provides a view at the complimentary angle to the direction of milling, thus revealing the appearance of the ‘front side’ of the sample being prepared. Without moving the stage, the ‘back side’ cannot be viewed with the SEM. For this reason, a symmetric series of milling steps is done on the back side, after confirming that a similar sequence gave good results as seen on the front side. Although some FIB‐SEM instruments facilitate simultaneous FIB milling and SEM imaging, the two processes cannot be individually optimised.

The FIB‐milled TEM lamellae are typically 10 µm wide and 10–20 µm deep, and several are made from each sample, as shown in Figure 6. The width and height of the windows are limited by the rigidity of the thin sheets of ice. Electrostatic forces during FIB milling may cause the thin lamellae to bend in the beam, resulting in ineffective milling or break‐through and complete loss. Therefore, several relatively narrow windows are made, interspersed with supporting material. The thickness can be adjusted according to the milling geometry, and is often 300–500 nm thick, which is appropriate for useful depth in a tomographic reconstruction. Reproducible production of very thin (∼100 nm) lamellae requires expertise, sample optimisation and good fortune. An experienced operator may be able to produce very thin lamellae between 50 and 150 nm, but the final thickness of the lamellae is limited by the semi‐arbitrarily electrostatics‐driven bending of the lamellae. The extent of bending depends on the flux of charged particles (ions and/or electrons) and the (biological) content of the lamella controlling the charge‐dissipation rate. When the flux is dissipated due to imaging between milling steps, the bending potentially changes. This can result in ineffective milling and can cause redeposition or even breakthrough and loss of the lamella. Although the SEM can be used simultaneously with the FIB, the image is not ideal, due to the required low‐kV/low‐current conditions needed to prevent electron‐beam damage. Therefore, the resulting milling step will be blind since it should not followed by SEM. As a result, the resulting thickness can only be determined afterwards, usually in the TEM.

The *in situ* determination of the thickness of the final lamella is challenging, especially in a ‘FIB view’ (edge‐on) image. In addition, the lamella thickness usually increases with the depth from the edge due to the FIB‐beam geometry. To some extent, the thickness can be made more uniform by milling both sides after slight tilting of the sample (Schaffer *et al*., 2017). Such a more‐parallel shape can be useful if the minimum possible thickness is desired. When using the CNB, rotating by 90º and tilting the stage can help to better assess the thickness by SEM. In general, when the minimum thickness is desired, one just tries to mill the specimen to be as thin as possible, hoping for a good result. An experienced operator will be successful much of the time. Since multiple lamellae can be made on one planchet or tube, an occasional failure is noncritical. The same milling strategy is applied to cells frozen on a TEM grid.

## Results

### TEM imaging

**Fig. 7:**
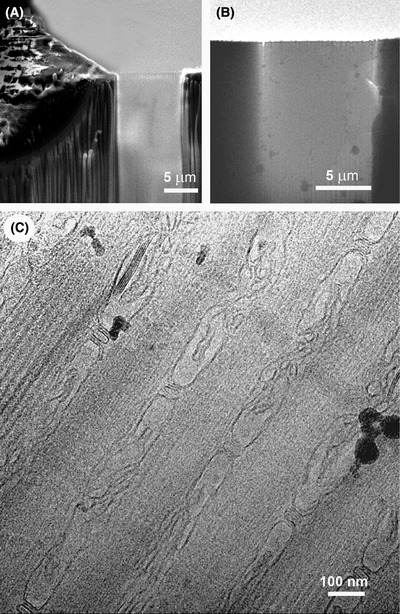
SEM and TEM results at Albany. In the SEM (A), a lamella suitable for TEM is ‘translucent’. TEM images (B, C) of a 200‐nm‐thick lamella have residual frost partcles (black spots) from the cryo‐transfers. A good‐quality TEM image (C) can be obtained, in this case of muscle tissue. All portions of this figure were assembled from portions of Figure 9 published in Hsieh *et al*. (2014).

Figure 7 shows an imaging sequence of the lamella in the FIB‐SEM, at low magnification in the TEM, and at normal TEM‐scale magnification. Good‐quality TEM imaging can be achieved from the FIB‐milled lamellae (Fig. 7C) (Marko *et al*., 2006; Hayles *et al*., 2010; Wagenknecht *et al*., 2015). Electron tomograms recorded from such samples have the potential of subtomogram averaging for study in *in situ* macromolecules (Wagenknecht *et al*., 2015).

### Temperature effects

Samples are normally stored under liquid nitrogen after high‐pressure freezing, and between operations. However, while in the cryo‐ultramicrotome, the temperature could rise to between –150°C and –160°C, and sometimes samples reach such temperatures during cryo‐transfer or on the cryo‐stage in the FIB‐SEM.

The coefficient of expansion for copper and ice are very different, and cooling down of both can causes the extrusion of a sample from a tube. The copper contracts while the ice expands (Schampers *et al*., 2014). The sample can be extruded from the copper tube to a height of 10–100 µm, as shown in Figure 5(A). Extrusion seems to have no effect on specimen preservation. Although currently not implemented in the workflow, the extrusion process could aid in reducing the number of transfers between different instruments, as it could make the cryo‐microtome superfluous. In any case, it is essential that the specimen be maintained at all times below the devitrification temperature (approximately –140°C).

### Cryo‐TSEM imaging and ice‐state evaluation

**Fig. 8:**
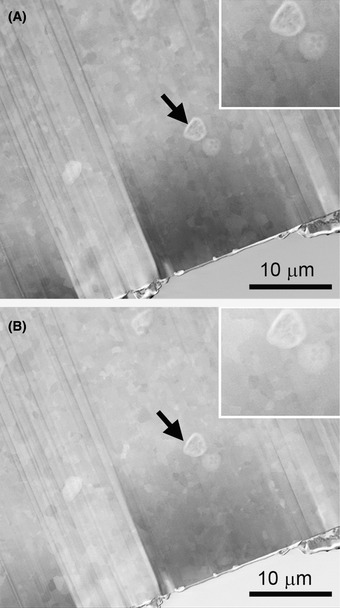
Cryo‐TSEM images of crystalline ice in a sample frozen in a copper tube. The specimen was mounted in the ‘cryo‐nano bench’ (CNB). Instead of tilting, which would change the working distance, the detector distance and the lateral position of the lamella with respect to the detector were altered. The rotation capability of the CNB is used to change the channeling conditions as a function of the crystallographic orientations, thus revealing any hexagonal ice crystallinity .

**Fig. 9:**
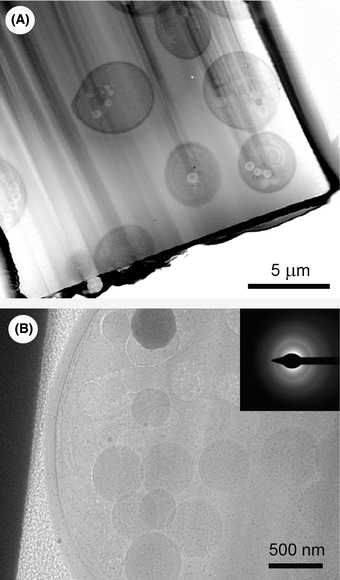
Results at Utrecht. Cryo‐TSEM (A), cryo‐TEM (B) images. The inset in (B) is a TEM diffraction pattern showing the faint sharp rings indicative of cubic ice.

Successful examples of the workflow in Utrecht have been presented by Hayles *et al*. (2010). It is useful to be able to distinguish whether the sample that has been frozen is in the vitreous state prior to transferring it to the TEM. To do this, a Transmission Scanning Electron Microscope (TSEM) detector was installed on the CNB. Consequently, TEM lamellae in copper‐tube samples mounted in the FIB‐SEM CNB stage can be evaluated by the TSEM detector (De Winter *et al*., 2013). Due to the low energy of the electron beam (<30 kV), good contrast is achieved despite the absence of staining (Figs. 8, 9A, C–D), and the vitreous state of the ice can be verified (De Winter *et al*., 2013). Briefly, the electron beam passes through the ice, and the interaction between the electrons and any ice crystals causes ‘diffraction contrast’, which varies as the beam tilts with respect to the sample. Thus, if patches in the sample are seen to vary strongly in brightness during small tilts, crystalline ice is present. Note that the cubic phase is likely not resolvable in the current cryo‐TSEM implementation due to the small ∼2 nm grain size of cubic ice. An example of the presence of crystalline hexagonal ice is shown in Figure 8. Several ice grains are clearly recognisable, and the variation in contrast indicates a different crystallographic orientation such that the relative intensity of neighbouring grains changes after tilting. In this case, we can conclude that the sample is not a candidate for cryo‐TEM. The correlation between crystallographic orientation and contrast has been confirmed by cryo‐transmission‐electron backscatter diffraction (cryo‐t‐EBSD) (De Winter *et al*., 2013).

An example of a cryo‐TSEM image without hexagonal ice crystals is shown in Figure 9(A). Cells are clearly visible, and ice crystals are not evident. However, after transferring to the cryo‐TEM, TEM diffraction indicated the presence of cubic ice (Fig. 9B), indicating that the temperature rose above –140°C. This could have occurred from handling with insufficiently cooled tweezers or from a poor thermal contact between the cooling block and the ferrule during any of the transfer stages in the process. Unfortunately, the cubic ice crystals cannot be resolved by the electron beam of a Nova Nanolab 600 FIB‐SEM instrument; therefore the position in the workflow where the devitrification occurred cannot be determined. Finding cubic ice in the lamellae is not common, but the presented example indicates the importance of checking the integrity of the lamella before proceeding with a time‐consuming process such as tomographic tilt‐series recording. The most reliable way of determining the presence of cubic ice is electron diffraction in the TEM (Dubochet *et al*., 1988).

## Discussion

As can be appreciated from the above, the use of cryo‐FIB‐SEM for preparation of samples for cryo‐TEM requires an experienced operator and dedicated equipment to achieve a high success rate. Many steps are involved, and each step is a potential source of difficulty. However, ongoing developments show that the field of cryo‐TEM preparation is maturing into a more commonly used high‐end technique (Kuba *et al*., 2020). Cryo‐FIB‐milled TEM lamellae provide unprecedented insights into native cellular environments. It has applicability to many scientific and biological questions. However, the making and handling of the fragile cryo‐FIB‐milled TEM lamellae is a delicate business, and the produced lamellae cannot be retained for archiving. Therefore, use of cryo‐FIB‐milled TEM lamellae is complimentary to a more ‘classic’ technique such as high‐pressure freezing followed by freeze‐substitution and conventional sectioning and staining. The spatial resolution of room‐temperature sections is limited to ∼2–5 nm, mainly by the stain granularity (Hsieh *et al*., 2006). The need for the higher resolution obtainable from vitreous frozen samples, exemplified by many recent macromolecular studies, is especially relevant when the goal is to study the macromolecules *in situ*. A wide range of interesting biological questions can only be answered by study of macromolecules *in situ* and in native state, which typically means in full‐thickness cells or in volumes of tissue. This is the province of cryo‐FIB‐SEM, as the only route to such information.

### Sample handling and transfer

We have shown here a variety of ways to handle cells and tissue for cryo‐FIB‐SEM, but common to all is protection from physical and thermal damage during cryo‐transfers. While maintenance of a temperature well below –140°C is essential, it is not especially difficult. However, avoidance of accumulation of frost during cryo‐transfer is more challenging. Fortunately, frost formed before the start of FIB‐milling is not a serious problem, since it can easily be removed before milling. We have found that brief ‘imaging’ of the bulk sample with a strong FIB beam (e.g. 5 nA) will quickly remove the frost, which is of low density compared to the bulk sample, which appears unaffected. Of course, strong irradiation is not appropriate for the final, thin lamellae, either in the FIB‐SEM or in the TEM.

The most critical cryo‐transfer is out of the FIB‐SEM and into the cryo‐TEM. The entire transfer can be split into two phases: (1) from the cryo‐FIB‐SEM chamber to the cryo‐TEM working station and (2) from the working station to the cryo‐TEM. The distinction is made between transferring the sample through vacuum or within LN2 versus transferring through ambient air. Although Gatan TEM cryoholder has a metal shutter and active cooling, we have experienced ice contamination during the prepumping loading sequence of the side‐entry cryo‐TEM. Modern cryo‐TEMs are often equipped with an automated loading system which greatly reduces the ice contamination risks during the transfer procedure.

Mechanical handling of the samples potentially leads to physical damage. When samples in copper tubes are held inside ferrules, or when samples in planchets are held in intermediate clamps, the samples are easier to protect from physical damage. The use of cryo‐transfer chambers that accommodate the specimen before and after milling under vacuum and at the required low temperature are essential. However, the prime source of frost before the TEM is the workstations where the (mounted) samples are moved to the TEM holder and then into the TEM. While some frost is always introduced during this step, it is not excessively bothersome in routine cryo‐TEM practice. By means of modified TEM holders or cartridges, no greater amount of handling is needed for cryo‐FIB lamellae than for other cryo‐TEM applications, and frost accumulation is manageable.

### Ion‐beam heating of the sample

Fortunately, the penetration depth of the Ga+ ions used in a FIB‐SEM is very shallow (∼10 nm; www.srim.org) when the milling is nearly parallel to the surface. The Ga+ ions sputter away the ice in this surface layer, but do not penetrate into the bulk tissue, so heating of the bulk does not occur. This was first experimentally confirmed (Marko *et al*., 2006), and then validated by cryo‐TEM tomography of FIB‐milled bacteria (Marko *et al*., 2007), showing that structure was undamaged as close as ∼10 nm from the milled surface.

### Electron beam damage

**Fig. 10:**
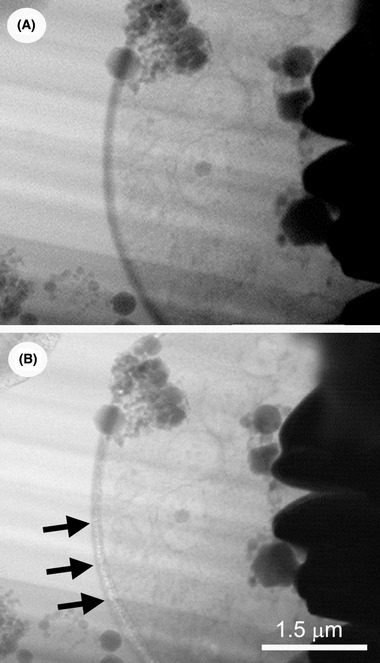
Electron‐beam radiation damage in the SEM. TSEM images before (A) and after (B) prolonged radiation. Arrows in (B) indicate splitting of a cell membrane.

The advantage of a FIB‐SEM instrument is that the progress and quality of FIB‐milling can be monitored and examined more closely with the electron beam than with the ion beam, the latter which inevitably mills even in imaging mode. The electron beam can also be used to compensate the positive charge built up during milling (Stokes *et al*., 2012). However, use of the electron beam must be judicious to avoid irradiation damage, which is especially critical as the lamellae approach TEM thinness. While the threshold for knock‐on damage is at a far higher energy (∼80 keV) than the typical 2–5 keV used in this work, and thermal effects are minimal with a cooled specimen and a moderate electron dose (Karuppasam *et al*., 2011), ice radiolysis (bond breakage with ‘bubbling’ from release of molecular hydrogen) can still occur (Fig. 10), as it does in cryo‐TEM at higher energy (Meents *et al*., 2010; Aronova *et al*., 2011; Carlson & Evans, 2012). Thus, the electron dose during cryo‐SEM and cryo‐TSEM has to be kept as low as possible. Use of scanning mode is an advantage here, since slow scanning may allow for ‘relaxation’ of the specimen as the beam moves on, or fast scanning might record a signal before damage occurs (De Winter *et al*., 2013). Of course, it is preferable that lamellae intended for TEM imaging are not be subjected to extensive SEM or cryo‐TSEM imaging.

### Stability

At times, FIB‐milling does not occur exactly where the pattern was set because either the sample has drifted or because the ion beam has drifted or has been deflected due to accumulated charge on the sample. Sample drift is rare because the temperature of the cryo‐stage is held very constant, but electrostatic charging of the sample certainly occurs. If the charge is not dissipated to ground, deflection of the ion beam is likely. Proximity to a good, grounded conductor, such as a grid bar or the remains of the specimen carrier (copper tube or aluminium planchet) helps. Metal deposition by sputter coating or by the gas‐injection system (with subsequent irradiation) may help (Hayles *et al*., 2007). Charge compensation by the electron beam may also help, although the required electron‐beam current could be a concern in regard to irradiation damage, even though the interaction volume is limited at low beam energy. Finally, once the lamella becomes quite thin, electrostatic charging can physically bend the lamella, unless it is well‐supported and not too wide.

### Future challenges

Two key challenges are identified: (1) sample handling and (2) localisation of the feature of interest.

#### Sample handling

Recent FIB‐SEM cryo‐transfer systems facilitate transfer of the sample under vacuum while maintaining the required low temperature. The samples can be stored under LN2 without being exposed to ambient atmosphere or contaminated dry nitrogen gas. Nevertheless, the sample may be exposed to room air or contaminated dry nitrogen gas when loading or unloading. TEM cryo‐transfer holders have proven to be efficient at accomplishing transfer with minimal frost, and at controlling the low temperature. The ideal way to handle cryo‐FIB‐milled samples would be to hold them in the TEM cryotransfer holder at the first transfer into the FIB‐SEM and at every transfer thereafter. This can be accomplished by mounting a TEM ‘goniometer’ on the FIB‐SEM chamber. An implementation of this has been described (Tsuchiya *et al*., 2015), and the Albany group plans such a system. However, this plan is only applicable to TEMs with a side‐entry specimen stage. This sort of TEM, while very common, does not include the most popular type of high‐end cryo‐TEM, having a cartridge‐type specimen holder. In that case, the cartridge itself is modified for use in both the FIB‐SEM and the TEM (Kuba *et al*., 2020).

#### Localisation of a feature of interest

The major challenge of cryo‐FIB preparation is to prepare the lamella to contain a particular region of interest in the bulk sample (tissue or whole cell). Correlative cryo‐fluorescent light microscopy has been employed for this purpose with considerable success (Arnold *et al*., 2016), mainly when the sample consists of cultured cells on a TEM grid. While the light microscopic *x*–*y* resolution is sometimes adequate, depending on the size of the labelled object and the microscope instrumentation, the *z*‐resolution is usually problematical. We can hope that correlative computational 3D mapping can assist. This can be complemented by SEM imaging of the surface of the sample as the FIB‐milling proceeds. Changes in localised charge on the sample face can make cellular components visible (Schertel *et al*., 2013; Kuba *et al*., 2020), and this kind of image may be of use in computational comparison with a 3D light microscopic volume.

Another possibility is the addition of a fluorescent light‐microscope inside the FIB‐SEM chamber or in the TEM (Wouters & Koerten, 1982; Agronskaia *et al*., 2008; Zonnevylle *et al*., 2013; Iijima *et al*., 2014; Gorelick *et al*., 2019; Loginov *et al*., 2019).

## Conclusions

The preparation of H‐bar cryo‐TEM lamellae from vitreous frozen whole cells or tissues is possible by cryo‐FIB milling, and we have shown a few methods for doing so. Three different approaches enable FIB‐milled cryo‐TEM lamellae: FIB‐thinning of single cells frozen on a TEM grid, TEM lift‐out and the H‐bar technique. Thinning cells on TEM grids has the advantage of fitting into existing workflows. Tremendous progress has been achieved since the technique was first published in 2006 (Marko *et al*., 2006), and the technique has evolved into a reliable route for detailed studies of macromolecular complexes within their native cellular environment (Marko *et al*., 2006; Rigort *et al*., 2012; Wagner *et al*., 2017). TEM lift‐out was long deemed ‘practically impossible’, but has emerged in two flavours: the classical lift‐out with a cold needle (Parmenter *et al*., 2016; Kuba *et al*., 2020; Parmenter & Nizamudeen, 2020), and using a cold ‘gripper’ (Schaffer *et al*., 2019). TEM lift‐out techniques also have the advantage of being part of existing workflows, in particular when loading into cryo‐TEM`. A benefit of both the lift‐out and H‐bar approaches is the ability to work with tissues, whole cells or cell pellets. The H‐bar approach is technically less demanding, in particularly if there were special planchets that would directly fit into existing cryo‐TEM stages

While cryo‐FIB preparation of cryo‐TEM lamellae is a demanding exercise, it is a worthwhile effort. The main challenges ahead are correlative approaches to predetermine the location of a specific site of interest, and increasing throughput, perhaps through automation (Kuba *et al*., 2020).
